# A classification of the verbal methods currently used to teach endoscopy

**DOI:** 10.1186/1472-6920-14-163

**Published:** 2014-08-09

**Authors:** Deng Mapiour, Michelle Prytula, Michael Moser

**Affiliations:** 1 Department of Surgery, University of Saskatchewan, 103 Hospital Drive, Saskatoon, Saskatchewan S7N 0W8, Canada; 2 Department of Educational Administration, College of Education, University of Saskatchewan, Saskatoon, Saskatchewan S7N 0W8, Canada

**Keywords:** Endoscopy, Gastroscopy, Colonoscopy, Skills, Verbal teaching

## Abstract

**Background:**

As endoscopy does not lend itself well to assisting or exposure by the teacher, most of the teaching is, by necessity, done verbally.

**Methods:**

The verbal teaching occurring during 19 colonoscopies and 14 gastroscopies was recorded by dictaphone and later transcribed. The resultant 53-page transcript was then analyzed using the Grounded Theory method. Teaching was compared between learners with less than one month versus more than one month of training and between teaching of colonoscopy versus gastroscopy.

**Results:**

The process of iterative review and repeated testing yielded 6 types of verbal teaching: demonstration by the teacher, motor instructions, broad tips/tricks/pointers, verbal feedback, questioning, and non-procedural information. Inter-rater agreement was excellent (Fleiss’s kappa = 0.76) between resident (DM), the non-medical educator (MP), and the medical teacher (MM). Overall, there was less non-procedural teaching (6.7% vs 23.7%, p = 0.01) and a trend towards more teaching moments per case (13.2 vs 7.9, p = 0.07) in the first month of the rotation compared to the later months. A greater proportion of the teaching for colonoscopy involved demonstration (13.7% vs. 2.7%, p = 0.040) and tips/tricks/pointers (26.6% vs. 12.4%, p = 0.012) compared to gastroscopy.

**Conclusions:**

We describe a means of categorizing verbal teaching in endoscopy that is simple and shows strong inter-rater agreement that will serve as a starting point for further studies aiming to improve how endoscopy is taught.

## Background

Endoscopy is a key diagnostic and therapeutic procedure in the repertoire of the gastroenterologist and gastrointestinal surgeon, and forms an important part of the training programs for both these specialties. Although there is much literature on the teaching [1-3] and the assessing of the teaching [4,5] that occurs in the operating room, particularly for laparoscopic surgery, the same cannot be said about the topic of teaching endoscopy. At a recent national meeting of program directors in General Surgery, an answer to the question “How should we be teaching endoscopy?” was not forthcoming. Although there exist excellent syllabuses [6], these describe very well the content that the learner is expected to learn during the fellowship, without describing how, exactly, the procedural teaching is to take place [7-9].

Simulators, both high-fidelity and low-fidelity, exist for laparoscopic surgery, in a variety of price ranges and with simultaneous application to several different surgical programs such as General Surgery, Gynecology, and Urology. Unfortunately, simulators for teaching endoscopy, which are generally specific to only one or two procedures, although validated [10], have not received the same degree of emphasis and still require verbal teaching and supervision for the learner to benefit from these educational experiences [11]. Furthermore, the costs associated with a colonoscopy-only simulator, for example, is prohibitive to most small to medium programs. Until the prices of these simulators come down considerably, the reality is that most programs will have to continue with the bedside teaching/apprenticeship model of teaching endoscopy.

Classic and time-honored approaches to teaching psychomotor skills date back to the late 1960 and early 1970’s, with the 7-step approach described by Simpson [12], which was subsequently expanded upon by Harrow [13] and Singer [14] and modified slightly for use in the ATLS educators course [15]. These include Perception, Set, Guided Response, Mechanism, Complex Overt Response, Adaptation, and Originating. The Guided Response step involves the learner performing the motor act under the supervision and guidance of a teacher. The teaching of endoscopy is somewhat unique in the teaching of psychomotor skills and is different from teaching in the OR, since it is simply not practical to ‘assist’ the learner with the endoscope. Even in the case of laparoscopic surgery, the teacher is able to intervene and expose or retract for the learner. The vast majority of the guidance and clinical teaching that occurs in the endoscopy suite is, as a result, necessarily *verbal*.

Similar to studies previously done on teaching in the Operating Room [16], our objective was to study the verbal teaching that occurs in the teaching of endoscopy, on the assumption that current teaching methods are effective at producing residents that are proficient and safe in endoscopy at the end of a 3-month rotation. We wished to identify different verbal methods in use, and develop a means of quantifying verbal teaching so that this may be used as a tool in future studies aimed at improving how we teach endoscopy. We also hypothesized that there are indeed differences between how endoscopy is taught in the early versus later phases of the 3-month rotation and also that there are differences between the teaching of gastroscopy and colonoscopy and that these differences would be apparent with the use of an appropriate classification.

## Methods

### Data collection

The verbal interactions during 19 colonoscopies and 14 gastroscopies were tape recorded from start to finish using a cassette recording device with sensitive microphone (Sony, BM-23, Tokyo). A total of 4 teachers and 5 residents were involved in the study. All teachers had greater than 5 years’ experience with teaching endoscopy and all residents were in their third year of residency, in the process of completing a 3-month endoscopy rotation. The authors were excluded from being teachers or learners in this study. In our program, the residents have virtually no exposure to endoscopy prior to the rotation such that the first month is very different from the next two. Residents on the endoscopy rotation are expected in the endoscopy suite at least four days per week and logbook records in previous years showed an average of 50 gastroscopies and 135 colonoscopies at the end of the three month rotation.

Two cases had almost no verbal interaction, both being near the end of the three month rotation, and presumably few verbal instructions were needed as a result. These were omitted from further analysis. The remaining 31 cases included 18 done during the first month and 13 from after the first month of the rotation. No identifying data was recorded except for the type of endoscopic procedure being performed (gastroscopy or colonoscopy) and which month of the 3-month endoscopy rotation the resident had attained at the time of the procedure.

The tape recordings were then transcribed, with names removed, using only “R:” and “A:” for resident and attending respectively, by DM, the co-author who was at the time a medical learner who had not yet done his endoscopy rotation and hence had no prior endoscopy experience. Transcription yielded 53 typed pages of interactions or 12000 words.

### Data analysis

The Grounded Theory method of Glaser and Strauss [17] was used to analyze the transcripts. This approach has been used in the past to look at verbal teaching in the operating room [18]. This is a validated, iterative process used in qualitative research, for testing and retesting hypotheses that explain the key issues in an interaction. The process was applied to the transcripts by the senior author and repeated until there was a complete iteration with no further changes. Once the 6 categories were identified, the non-medical educator (MP) went through the transcript one more time, highlighting the teaching ‘moments’. A random sample of 50 ‘moments’ was selected by someone with neither a medical nor and education background to assess interobserver agreement between the learner (DM), the educator (MP), and the clinical teacher (MM). A sample size of 50 was chosen by the three co-authors; the reasoning was that this represented over 10% of the teaching moments and also this would allow for review by each of the reviewers in one seating. Fleiss’s Kappa was used to quantify this agreement between our three observers. The Mann–Whitney U test was used for all other analyses.

The number of teaching moments of each type was then tabulated for each endoscopic procedure and standardized by dividing the number of teaching moments in each category by the total number of teaching moments for that procedure, to account for the fact that some procedures were longer and more complex than others.

Our study was approved by the Behavioral Research Ethics Board of the University of Saskatchewan. In each case the patient, resident, and faculty teacher all gave consent for their inclusion in the study. No teacher or learner refused to be in this study once approached and only two patients refused, in each case this was likely due to a poor understanding of the study and/or recording process.

## Results

**Figure 1:**
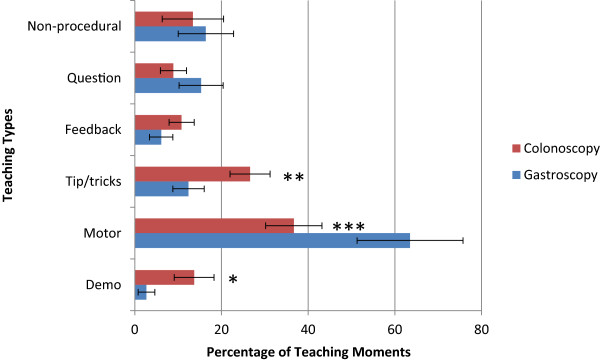
Comparison of teaching types used in teaching colonoscopy versus gastroscopy.

**Figure 2:**
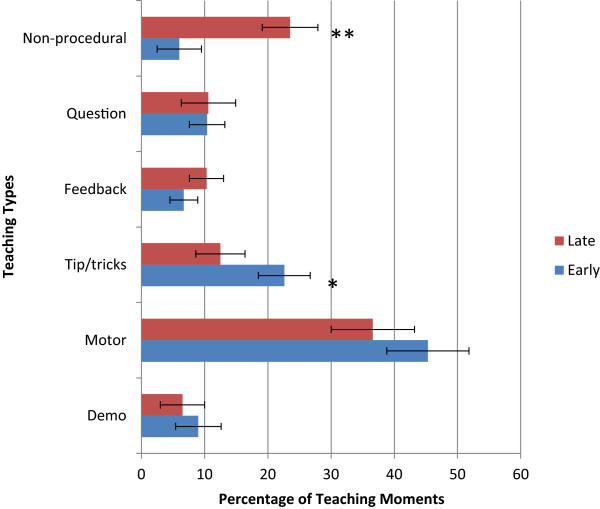
A comparison of the teaching types used for the learner’s first month on the rotation versus the later months.

There were a total of 384 teaching moments identified in the 19 colonoscopies and 14 gastroscopies making up the 53-page, 12000 word transcript. The first scan of the transcript yielded 19 possible categories of teaching moments and after 5 complete iterations of the 53-page transcript, six categories remained (Table 1), with no further changes on the final iteration.The distribution of the different types of teaching used is significantly different (Figure 1). There was a trend towards more teaching moments in colonoscopy than gastroscopy (15.3 vs. 9.6, p = 0.12). Motor instructions were used more in the teaching of gastroscopy than in colonoscopy (p = 0.0001). Demonstration and “tips, tricks, pointers” were used more in the teaching of colonoscopy versus gastroscopy (p = 0.04 and 0.012 respectively).The distribution of the different types of teaching used also differed between the first month of instruction and the later months of instruction (Figure 2). There was a trend towards more teaching moments per case in the first month (13.2 vs. 7.9, p = 0.07). The first month was characterized by markedly less non-procedural teaching in the first month (p = 0.01) and a trend towards more “tips, tricks, and pointers” (p = 0.07) compared to later stages of training. Otherwise, there were similar amounts of demonstration, motor instructions, and feedback.

**Table 1:** Examples of the 6 categories of verbal teaching methods

**Category**	**Examples**
Demonstration with verbal narrative	“So I turn the big wheel to try to look around that corner, all the while ensuring continued torque on the scope”
Motor instruction	“… keep it centered. Now, look up. Look up again by using the big dial. … tap, tap on the suction …”
	“stay out of that stool.”
Tips/tricks/pointers	“… the trick to any colon is you’ve got to negotiate the sigmoid colon without too much insufflation. It makes all the difference.”
Feedback	“Good. Well done”
Questioning	“What do you think of the stomach lining over there?”
Non-procedural information	“If we don’t find the bleeding here, the next step should be a nuclear medicine scan”

The interobserver agreement between the learner, the educator, and the clinical teacher was excellent, with a Fleiss’s Kappa of 0.76, even though the educator had no formal medical background and the learner had no formal education background. Complete agreement by all three observers in terms of classification was seen for 82% of the teaching moments.

## Discussion and conclusions

In order to optimize the teaching of endoscopy, we first have to understand how endoscopy is currently taught. Because of the nature of the endoscopic procedure, we recognize that most of the teaching is done verbally. In this study, through the use of the grounded theory method, we arrived at a classification of the various verbal methods that are used to teach endoscopy, that has excellent interobserver agreement that may form a baseline for future work directed at optimizing how endoscopy is taught verbally.

Our classification has discriminant ability in that differences were noted in the distribution of the various teaching types depending on whether it was gastroscopy or colonoscopy that was being taught. Also, our system was sensitive to the fact that teachers seem to adapt their verbal teaching styles based on whether the learner is in the early stages of the rotation or the later stages. In the early stages of learning, as expected, the teacher was more likely to teach by demonstration and direct, detailed instruction. Later on in the rotation, there was increased discussion of non-procedural information and a trend toward more questioning of the learner; to do so in the early stages, while the learner was still getting familiar with the instrument would have been too distracting [19]. Likewise, it makes intuitive sense that more broad tips and tricks were offered up by the teacher in the teaching of colonoscopy which is more complex and lengthy than gastroscopy. Finally, our classification system demonstrated substantial inter-rater reliability with very strong agreement between our medical teacher, our resident learner, and our non-medical teacher (kappa = 0.76).

Although some previous works have studied the evaluation of the learner [20,21], our classification provides an objective way of measuring how the *teacher* verbally teaches endoscopy.

The number of categories in our classification finds itself in between that of previous studies looking at verbal teaching in the operating room. One study [16] analyzed verbal communication in the OR and classified the verbal content into one of four broad categories, with each of the categories having between 3 and 9 subcategories, yielding 23 different subcategories compared to our six. They noted interobserver agreement of 70% in keeping with what we found. Another study [18] using the Grounded Theory method arrived at four categories; although these were useful for the purposes of their study, each category was very broad; nothing about the four categories gives much in the way of specifics to a teacher looking to improve his or her verbal teaching.

Our classification provides a practical and simple classification with a reasonable number of categories that should be helpful in determining which of the types of verbal teaching is most effective for which procedure and at what stage in the learning. When our results were presented at a Departmental Research Day, simply hearing about the different verbal methods used to teach endoscopy caused several teachers to think about and change the way they teach endoscopy and to incorporate some of the teaching types they had not previously thought of. It will be interesting to see how awareness of these teaching types changes the way in which endoscopy is taught at our institution.

Our study was limited by the fact that the teachers were all surgeons; teachers from gastroenterology may have a very different approach to teaching endoscopy. Anectdotally, surgery-based teachers of endoscopy notice a difference between teaching endoscopy to a learner from a surgical program versus one from a gastroenterology program. Likewise, variation may exist between different institutions. Our study incorporated a relatively small number of teachers [4], however, these teachers have high volumes of experience in teaching endoscopy and all four of these teachers are highly regarded in end-of-rotation evaluations by the residents.

The Hawthorne Effect, whereby a teacher may modify or improve his or her teaching behaviour as a result of knowing they are being studied, has no doubt biased our study, but since our objective was to identify verbal methods of teaching endoscopy, this was a helpful bias.

Future studies are needed and are underway. In particular, we will be looking at how the distribution of teaching types will change once the different types of teaching moments identified in this paper are reinforced among our faculty teachers. In a separate study at our centre, a daily and weekly rating scale for the learner is being developed and validated. This will be useful in a future study to see how changing the teachers’ approaches to teaching endoscopy changes the rate at which the various ratings are achieved by the learners. Anecdotally, colleagues are already noticing that the residents are attaining ‘endoscopic comfort’ sooner as a result of an awareness of our classification of verbal methods of teaching endoscopy.

## Competing interests

There are no conflicts of interest.

## Authors’ contributions

DM carried out the study design, carried out the data acquisition, participated in data analysis and manuscript preparation. MP carried out study design, participated in data analysis, and carried out the manuscript preparation. MM carried out the study design, participated in data analysis and manuscript preparation. All authors read and approved the final manuscript.

## Authors’ information

DM is a General Surgery resident with an interest in medical education and international surgery ever since he was a medical student.

MP is a former elementary school teacher and principal, who is now Dean of the College of Education. She maintains a serious interest in (tongue in cheek) “the similarities between teaching children and the training of new doctors”.

MM is an academic General Surgeon with over 12 years’ experience teaching medical students and residents, including being residency program director for the General Surgery program. He also devotes time to working with the biomedical engineers to improve upon the design of the modern-day colonoscope.

## Pre-publication history

The pre-publication history for this paper can be accessed here:

http://www.biomedcentral.com/1472-6920/14/163/prepub
